# Outcomes of a 24-month study of patients with HIV with Cryptococcal meningitis on high-dose fluconazole induction in Abidjan, Côte d'Ivoire, between 2012 and 2016

**DOI:** 10.1016/j.ijregi.2025.100691

**Published:** 2025-06-18

**Authors:** Affoué Gisèle Kouakou, Raoul Moh, Frédéric Nogbou Ello, Constant Ozigré, Salif Diawara, Hermann N’Guessan Faitey, Serge Niangoran, Alain N’douba Kassi, Chrysostome Mossou, Fulgence Kondo Kassi, Aristophane Tanon, Serge Paul Eholié

**Affiliations:** 1 Unité de Formation et de Recherche des Sciences Médicales, Université Félix Houphouët Boigny, Abidjan, Côte D'Ivoire; 2 Service des Maladies Infectieuses et Tropicales (Department of Infectious and Tropical Diseases), Treichville University Hospital, Abidjan, Côte D'Ivoire; 3 PACCI programme, ANRS Côte d’Ivoire Centre, Abidjan, Côte D'Ivoire; 4 Centre de Diagnostic et de Recherche sur le Sida et les Maladies Opportunistes, Centre Hospitalier Universitaire de Treichville, Abidjan, Côte d’Ivoire

**Keywords:** Cryptococcal meningitis, HIV/AIDS, Fluconazole, Long-term outcomes, Côte d'Ivoire

## Abstract

**Objectives:**

Cryptococcal meningitis (CM) is a common cause of meningitis in patients with AIDS in sub-Saharan Africa, with a mortality rate of over 50% at 10 weeks. The preferred treatment in resource-limited countries without access to amphotericin B or 5-fluorocytosine is high-dose fluconazole (FCZ). However, survival and factors associated with mortality after completion of FCZ-based treatment are not well known. To assess the outcomes at 24 months of patients with HIV with CM who have completed the initial FCZ treatment.

**Methods:**

Retrospective cohort study of adult patients with HIV with CM on oral FCZ 1200 mg/day induction, having completed 10 weeks of specific treatment between January 2012 and December 2016. The survival probability (not lost to follow-up or death) at 24 months was determined and the risk factors associated with death were identified using the Cox proportional hazard model.

**Results:**

Thirty-one (31) patients were enrolled from a total of 82. The median age was 42 years (38-44). Overall, 58% of the patients were female (n = 18) and 50% (14/28) were antiretroviral therapy experienced. The following outcomes were observed after 24 months of follow-up: 13 patients (41.9%) were lost to follow-up, 12 (38.7%) were still alive, 6 (19.3%) died, and 5 (16.1%) relapsed. The mortality rate was reduced by 77% where the clusters of differentiation 4 count was less than 100 cells/mm^3^, with adjustments for length of hospitalization and history of morbidities.

**Conclusions:**

Long-term survival among patients with HIV with CM was poor. Interventions to strengthen linkage to HIV treatment and care and continuation of secondary fluconazole prophylaxis are critical.

## Highlights

• High loss to follow-up rate after initial antifungal treatment.
• Most deaths occurred within the first 6 months of follow-up.
• Relapses of cryptococcal meningitis were the main cause of death.
• Strengthening therapeutic education is necessary to improve the long-term prognosis.

## Introduction

Cryptococcal meningitis (CM) is a common cause of meningitis among adults with HIV in sub-Saharan Africa. In 2014, the region accounted for an estimated 162,500 cases (73% of the global total) and 135,900 deaths (75% of the world total) [1]. The World Health Organization recommends a first-line treatment consisting of amphotericin B deoxycholate (AMB) and 5-fluorocytosine (5-FC), followed by fluconazole (FCZ) [2]. However, the use of AMB is limited in many resource-poor settings due to its toxicity, high cost, and the challenges of maintaining a cold chain. This is particularly problematic in Côte d’Ivoire, where 5-FC is unavailable [[3], [4], [5]]. As a result, the treatment often defaults to FCZ monotherapy (1200 mg daily), which raises concerns about the potential development of resistance to this antifungal. Indeed, resistance to FCZ is becoming a significant concern globally [6].

In developed countries, premature death within the first 10 weeks ranges from 10 to 25%, whereas in resource-limited countries, it spikes to 60-70% [1,5]. Long-term outcomes of HIV-related CM are only documented in a limited number of studies, and their findings are not universally applicable. Studies that have examined survival rates in relation to treatment suggest that mortality tends to be lower when AMB is included in the induction phase of treatment [7,8]. Moreover, the long-term benefits of induction therapy with fluconazole remain poorly understood in Africa, despite its widespread use. This study aims to explore the long-term outcomes of patients who have undergone induction therapy with high-dose fluconazole for CM and to identify the factors influencing these outcomes.

## Materials and methods

### Study type and population

This retrospective cohort study focuses on adults with HIV diagnosed with CM who underwent a high-dose FCZ induction phase. The participants completed their initial 10-week treatment regimen between January 2012 and December 2016 at the Service des Maladies Infectieuses et Tropicales (SMIT) in Abidjan, a key referral center for the management of HIV and infectious diseases. The facility includes a 68-bed hospital block and accepts referrals from tertiary health facilities.

Exclusion criteria were patients who died before completing the first-line treatment and those who received less than 1200 mg of FCZ during the induction phase.

### Diagnostic process

CM diagnosis was established through the identification of budding encapsulated yeasts in cerebrospinal fluid (CSF) samples, using Indian ink staining, positive cultures on Sabouraud medium, or detection of cryptococcal antigen (CrAg). CSF samples were analyzed at the laboratory of the Centre de Diagnostic et de Recherche sur le Sida et Maladies Opportunistes (CeDReS) at Treichville University Hospital.

For antigen detection, agglutination tests were conducted using the PASTOREX™ CRYPTO PLUS kit (BIO-RAD, France) as per the manufacturer’s instructions. Cultures were aseptically grown on Sabouraud chloramphenicol medium and incubated for 2-7 days at 37°C. Positive cultures were further tested for urease secretion using indole and the strains’ biochemical characteristics were assessed using ID32C galleries to confirm cryptococcus identity.

### Treatment procedure

At the time of the study, the national recommendations for CM treatment specified an induction phase involving oral FCZ at 1200 mg daily, either alone or combined with flucytosine (5-FC) for 2 weeks. This was followed by an 8-week consolidation phase with FCZ at 800 mg daily [2,3]. In the maintenance phase, FCZ 200 mg was administered daily until the patient's immunity was restored [2,3]. Additionally, therapeutic lumbar punctures were performed daily to remove 30 ml of fluid when intracranial pressure exceeded 35 cm H₂O. If the intracranial pressure was between 25 and 35 cm H₂O, 20 ml was removed daily until the pressure decreased below 25 cm H₂O [2,3].

Antiretroviral therapy (ART) commenced 4 weeks after starting antifungal therapy for CM, with the preferred regimen being a fixed-dose combination of tenofovir, lamivudine, and efavirenz [9].

### Data collection

Initially, an inventory was taken of patient files that met the inclusion criteria, based on hospital registration documents and medical records from the SMIT. Subsequently, data on disease progression were gathered using HIV monitoring traceability tools, including the SMIT electronic database, and direct communication (telephone calls and/or emails) with patients treated outside SMIT.

These methods provided comprehensive socio-demographic, clinical/biological, treatment, and outcome data, supplemented by information collected through standardized questionnaires.

### Definition of terms

CM case: A diagnosis is confirmed by the detection of spherical or oval encapsulated yeasts in the patient’s CSF, ranging from 3 to 10 μm through the Indian ink test. The presence of these yeasts indicates *Cryptococcus neoformans*, and meet the international criteria for the diagnosis of invasive mycoses [10].

CM relapse: The recurrence of symptoms accompanied by the recovery of viable organisms from previously sterile CSF [3].

Lost to follow-up: Defined as when the latest available information is dated more than 3 months since the last scheduled appointment.

### Statistical analysis

Data were entered using Epi Data software version 3.1 and analyzed with R Studio software version 1.043.

Categorical variables were summarized using frequencies and percentages, while continuous variables were described using means and standard deviations. The data were converted to ‘survival’ format, meaning time-to-event data. The completion of the 10-week CM treatment marked the entry point (time 0) for each participant, with censoring occurring at 2 years from this point or at the last observed time. Time accumulated was measured in person-years of follow-up. Survival trends over the 24 months were estimated using a Kaplan-Meier curve. Cox’s univariate and multivariate proportional hazards models were utilized to explore factors associated with the risk of death or loss to follow-up at 24 months, defined as the absence of updated vital status. Variables with a likelihood ratio test *P*-value of 0.2 or less in the univariate analysis were included in the multivariable Cox regression. The final model was developed through a stepwise selection process, retaining variables significant at a *P*-value of.05.

## Results

**Figure 1:**
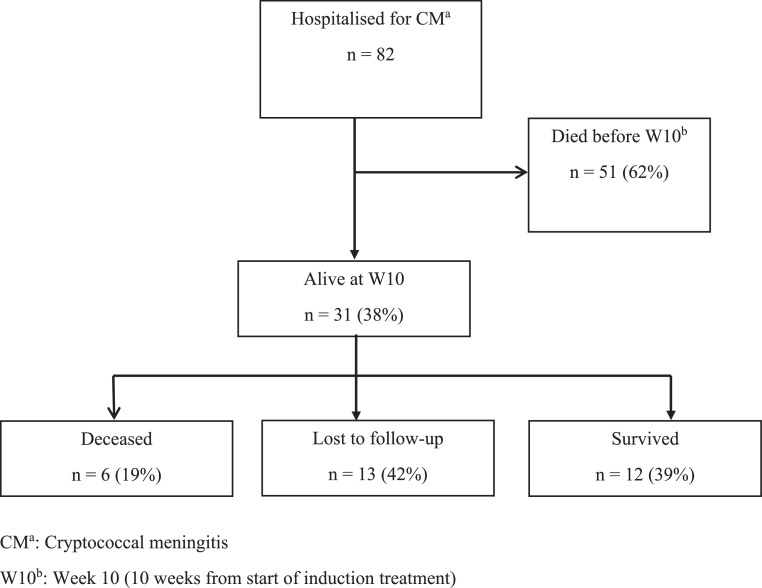
Diagram showing the flow of patients with CM hospitalized at the Service desMaladies Infectieuses et Tropicales (SMIT), Abidjan 2012-2016.

During the study period, 82 patients were hospitalized for CM treatment. The initial regimen included FCZ at 1200 mg for the 2-week induction phase, followed by 800 mg for the subsequent 8 weeks, either as monotherapy or combined with 5-FC. Of these, 31 patients (38%) completed the initial 10-week phase (W10) and were included in the study analysis (Figure 1).

CM^a^: Cryptococcal meningitis; W10^b^: week 10 (10 weeks from start of induction treatment).

### Socio-demographic, clinical, biological, and therapeutic characteristics at the time of CM diagnosis (Table 1)

The median age of the study participants was 42 years (range 38-44), with 58% being female (n = 18). The primary opportunistic infections present at diagnosis were tuberculosis in 14 of 29 patients (48%) and cerebral toxoplasmosis in 10 of 29 patients (34%). All participants were HIV-1 positive, and half of them (14/28) had previously received ART. The median cluster of differentiation (CD4) cell count was 88 cells/mm³ (range 15-173).

Clinically, the onset of symptoms was subacute in 77% of cases. Meningoencephalitis was diagnosed in 45% of patients, with a Glasgow Coma Scale score of <12 in 3 of 14 cases. CSF analysis indicated a median cytology of 50 cells/mm³, ranging from 11 to 150 cells/mm³. The Indian ink and CrAg were utilized in all cases (100%), aiding in the detection of positive cultures in 12 cases (39%).

Regarding treatment, fluconazole was administered as monotherapy in 45% of cases (14/31) and in combination with 5-FC in 55% of cases (17/31). In the maintenance phase, 86% of patients received 200 mg of FCZ, while 14% received 400 mg.

### Patient progression data at 24 months

**Figure 2:**
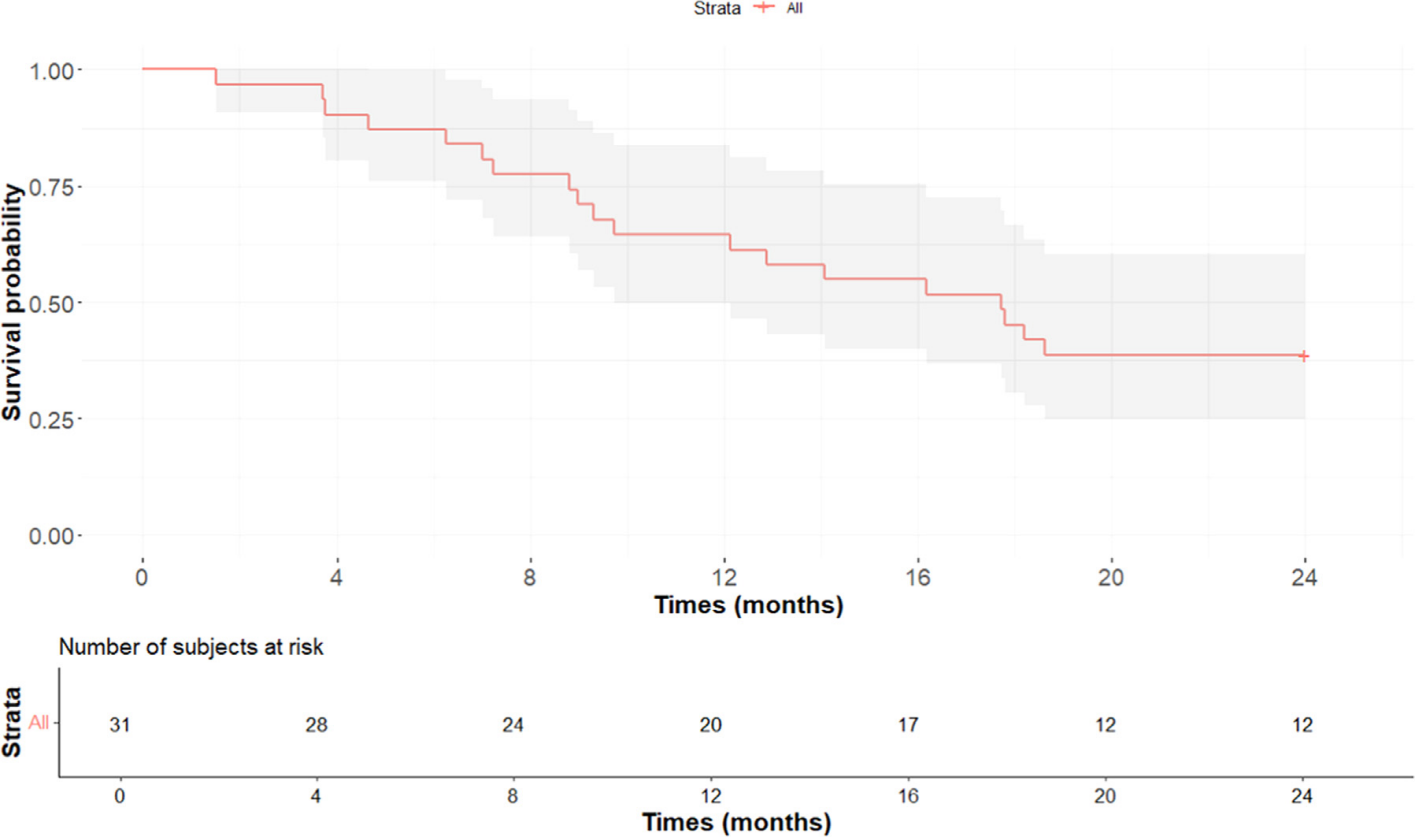
Survival probability over time (months) and number of subjects at risk. Cumulative probability of survival in death or loss to follow-up.

Outcomes at 24 months following the completion of the W10 treatment phase included: 13 patients (42%) reported as lost to follow-up, 12 patients (39%) still alive, six patients (19%) deceased, and five patients (16%) had relapsed (Table 2). The rate of loss to follow-up and mortality per 100 person-years was 47 patients after 40.5 person-years of follow-up. Almost all deaths and losses to follow-up occurred within the first 18 months. The survival probability at 12 months—defined as still being alive, free of the pathology concerned, and not lost to follow-up—was 64.5% (95% CI: 49.7-83.8%), which then decreased to 38.7% at 24 months (95% CI: 24.9-60.3%) (Figure 2).

**Table 1:** Socio-demographic, clinical, biological and treatment data for Cryptococcal meningitis survivors at week 10, Department of Infectious and Tropical Diseases (SMIT), Abidjan 2012-2016, n = 31.

***Sociodemographic***	
Age (years), median (IQR)	42 (38-44)
Female, n (%)	18 (58)
***Clinical***	
**Existence of major opportunistic infections and comorbidities (n = 29), n (%)**	
Tuberculosis, n (%)	14 (48)
Toxoplasmosis, n (%)	10 (34)
**Signs - type of onset**	
Subacute, n (%)	24 (77)
Acute, n (%)	07 (23)
Meningococcal disease, n (%)	17 (55)
Meningoencephalitis, n (%)	14 (45)
Glasgow score <12 (n = 14), n (%)	03 (21)
***Biological***	
**HIV data**	
HIV-1, n (%)	31 (100)
Clusters of differentiation 4 count (cell/mm^3^), median (IQR)	88 (15-173)
**Cerebrospinal fluid analysis**	
White blood cell count, (n = 29), median (IQR)	50 (11-145)
Lymphocyte count >50%, n (%)	28 (90)
Positive India ink, n (%)	31 (100)
Positive cryptococcal antigen, n (%)	31 (100)
Positive culture, n (%)	12 (39)
***Treatment***	
Treatment strategy at induction phase	
Monotherapy (Fluconazole), n (%)	14 (45)
Dual therapy (Fluconazole + 5-fluorouracile), n (%)	17 (55)
……Subtractive lumbar punctures, median (IQR)	03 (2-6)
Antiretroviral experience^c^	
yes, n (%)	14 (50)

IQR, interquartile range.

^a^Antiretroviral experience: n = 28.

**Table 2:** Progression data for Cryptococcal meningitis survivors at week 10, Department of Infectious and Tropical Diseases (SMIT), Abidjan 2012-2016.

**Fluconazole dose at maintenance phase**	
Fluconazole 200 mg	15 (48.4)
Fluconazole 400 mg	16 (51.6)
**Outcome at 24 months, n (%)**	
Lost to follow-up	13 (42)
Alive	12 (39)
Deceased	6 (19)
Relapsed	5 (16)

### Factors related to death or loss to follow-up at 24 months

The only variable significantly associated with the risk of death or loss to follow-up was the CD4 count. Patients with a CD4 count greater than 100 cells/mm³ had a 77% reduced risk of a fatal outcome, after adjusting for the length of hospitalization and history of morbidities (Table 3).

**Table 3:** Factors associated with death or loss to follow-up at 24 months, Cryptococcal meningitis survivors at week 10, Department of Infectious and Tropical Diseases (SMIT), Abidjan 2012-2016.

	Univariable			Multivariable		
	Adjusted hazard ratio	95% confidence interval	*P*-value	Adjusted hazard ratio	95% confidence interval	*P*-value
Age	1.03	(0.97-1.10)	0.335			
History (yes or no)	2.24	(0.80-6.24)	0.124	2.24	(0.78-6.42)	0.132
Protein concentration (≥0.66 vs <0.66)	0.4	(0.14-1.12)	0.082			
Hemoglobinemia (≥10 vs <10)	0.35	(0.13-1.00)	0.050			
ASAT (>30 vs ≤30)	2.17	(0.63-7.49)	0.218			
ALAT (>35 vs ≤35)	1.95	(0.74-5.15)	0.176			
Creatine clearance in ml/min (≥60 vs <60)	3.37	(0.44-.80)	0.242			
Length of hospitalization (> 15 vs ≤ 15)	0.23	(0.07-0.70)	0.009	0.38	(0.12-1.20)	0.100
Clusters of differentiation 4 count (≥100 vs <100)	0.19	(0.06-0.68)	0.010	0.23	(0.06-0.84)	0.026

## Discussion

CM is a significant cause of meningitis among patients with HIV in sub-Saharan Africa, with 162,500 cases and 135,900 deaths reported in 2014 [1]. Aoussi *et al.* [11] reported a CM prevalence of 2.6% in Côte d'Ivoire hospitals, with an estimated mortality rate of 41.2%. This retrospective analysis of survival data for HIV patients with CM presents quite discouraging long-term outcomes. The survival rate at 24 months from the end of the initial treatment was only 38.7%. There were 13 of 31 patients (42%) lost to follow-up, and the fatality rate following initial hospitalization was 19% (6/31). Notably, fewer fatal outcomes occurred among patients with a CD4 count greater than 100 cells/mm³ at the time of CM diagnosis.

This study contributes valuable long-term survival data for HIV patients with CM from West Africa, a region less frequently studied compared to Eastern and Southern Africa, where most CM research has been conducted. Survival rates reported in various studies from these regions remain low, typically less than 50% [[12], [13], [14]]. Literature suggests that significant factors associated with fatality include altered mental status and/or the presence of seizures at the time of initial CM diagnosis [5,12,13] as well as rehospitalization [15]. In contrast, our study did not find these factors, although 45% of the patients had meningoencephalitis, associated with coma in three cases, and all five patients who relapsed subsequently died.

However, regular follow-up visits, adherence to ART, and secondary prophylaxis with FCZ, along with weight gain, appear to positively impact survival [13,15]. In this study, the only factor clearly associated with reduced fatality was a CD4 count greater than 100 cells/mm³, after adjusting for the length of hospitalization and history of morbidities.

Furthermore, a cost-effective strategy that offers survival benefits involves sub-clinical management of the disease through screening for cryptococcal antigenemia and pre-emptive antifungal treatment, as noted by Butler *et al.* [16,17].

Overall, the long-term prognosis for CM in symptomatic HIV patients remains poor in sub-Saharan Africa, with potential 1-year mortality rates as high as 78%, according to Pasquier *et al.*’s [7] literature review. The relatively low mortality rate of 19% at 24 months observed in this study likely reflects the impact of patients recorded as lost to follow-up, who may have died. Since this study addresses both loss to follow-up and death during follow-up, the high proportion of patients lost to follow-up negatively influences the observed survival rate.

Although mortality rates varied across studies, deaths predominantly occurred within 6 months of diagnosing CM [6,12]. The results of this study align with the literature, showing a median follow-up time of 5.5 months for patients who subsequently died.

Strategies to improve retention in HIV follow-up cohorts are crucial. According to Rosen *et al.* [18], the top HIV care programs in sub-Saharan Africa retained 85% of their patients after 2 years of follow-up. Attrition rates were lower among highly immunocompromised patients, and routine patient tracing was linked to better retention [19].

However, pursuing patients who are lost to follow-up presents challenges in routine care.

HIV care programs need to ensure patient identification, accessibility, and continued availability of antiretrovirals. In Côte d’Ivoire, the system for identifying patients is not standardized at the national level, and effective patient tracing mechanisms, such as a unique national identification number, phone number, email, or postal address, are necessary for this specific population.

Given these challenges, priorities have been set to improve the prognosis of HIV-associated CM in resource-limited settings. One key recommendation is the early detection of CM through serum CrAg in patients with HIV presenting at advanced stages of infection (CD4 T-cell count <100 cells/μl), coupled with preventive antifungal treatment for asymptomatic CrAg-positive patients [2,4,17]. Moreover, the availability of essential services such as HIV self-testing and routine antiretroviral treatment supports community-based HIV screening and early management [20].

These guidelines have been adopted as national recommendations in Côte d'Ivoire and have been implemented since 2019. The establishment of a national registry for patients with CM would also facilitate more comprehensive follow-up data.

This study has several limitations that should be acknowledged: the small size of the study population, the limited generalizability of the results due to its single-center focus, the high number of patients lost to follow-up, which could introduce an information bias, and factors such as adherence to antifungal treatment and the incidence of immune reconstitution inflammatory syndrome (IRIS) that were not documented.

## Conclusion

This study demonstrates that patients remain at risk of mortality after completing treatment for CM, particularly in the 6 months following treatment completion. Enhanced outpatient monitoring and improved education about treatment during follow-up are essential for HIV patients with CM. Promoting early HIV testing and adopting a differentiated care model could also improve the medium- and long-term prognosis for these patients. Further research involving a larger sample size, through multicenter and/or qualitative studies, is necessary to gain more precise insights into the determinants of long-term outcomes.

## Declarations of competing interest

The authors have no competing interests to declare.
